# Cardiovascular Health at the Intersection of Race and Gender in Medicare Fee for Service

**DOI:** 10.1001/jamahealthforum.2025.3014

**Published:** 2025-08-22

**Authors:** Gray Babbs, Kendra Offiaeli, Jaclyn M. W. Hughto, Landon D. Hughes, Theresa I. Shireman, David J. Meyers

**Affiliations:** 1 Department of Health Services, Policy, and Practice, Brown University School of Public Health, Providence, Rhode Island; 2 Center for Gerontology & Healthcare Research, Brown University School of Public Health, Providence, Rhode Island; 3 Department of Behavioral and Social Sciences, Brown University School of Public Health, Providence, Rhode Island; 4 Department of Epidemiology, Brown University School of Public Health, Providence, Rhode Island; 5 Center for Health Promotion and Health Equity, Brown University School of Public Health, Providence, Rhode Island; 6 Department of Population Medicine, Harvard Pilgrim Health Care Institute, Boston, Massachusetts; 7 Department of Epidemiology, Harvard T.H. Chan School of Public Health, Boston, Massachusetts; 8 Center for Advancing Health Policy through Research, Brown University School of Public Health, Providence, Rhode Island

## Abstract

**Importance:**

Transgender and gender diverse (TGD) people have significantly higher rates of cardiovascular-related conditions than cisgender people, and Black and Hispanic people have higher rates of cardiovascular-related conditions than non-Hispanic White people. However, little is known about the prevalence of cardiovascular-related conditions among racial and ethnic subgroups of TGD people.

**Objective:**

To compare the prevalence of cardiovascular-related conditions across racial and ethnic groups for TGD and cisgender people using quantitative intersectional methods.

**Design, Setting, and Participants:**

Medicare enrollment and claims data were used from TGD and cisgender beneficiaries from 2011 to 2020. Using an established algorithm, likely TGD people were identified based on their diagnosis codes and care utilization. The 10 nearest-neighbor cisgender matches for each TGD beneficiary were identified based on propensity scores estimated from the original basis of eligibility, years of enrollment, age, and hospital service area.

**Exposure:**

Race, ethnicity, and gender modality (TGD and cisgender). These data were analyzed from November 7, 2023, to October 31, 2024.

**Main Outcomes and Measures:**

Rate of cardiovascular-related conditions (peripheral vascular disease, congestive heart failure, diabetes, hypertension, and chronic obstructive pulmonary disease) among Asian and Pacific Islander, Black, and Hispanic TGD beneficiaries compared with non-Hispanic White cisgender counterparts using generalized estimating equations, cardiovascular diseases and their risk factors. Attributable proportions for TGD Asian and Pacific Islander, Black, and Hispanic beneficiaries were calculated.

**Results:**

Of the 36 004 TGD beneficiaries, 714 Asian and Pacific Islander (2%), 4518 Black (13%), and Hispanic 2545 (7%) had higher rates of cardiovascular-related conditions than 28 227 non-Hispanic White (78%) beneficiaries and higher than the 323 613 cisgender beneficiaries (5981 Asian and Pacific Islander [2%]; 40 781 Black [13%]; 22 417 Hispanic [7%]; 254 434 White [79%]). Black TGD beneficiaries had a 74% higher prevalence of peripheral vascular disease, 76% higher prevalence of congestive heart failure, and 50% higher prevalence of diabetes than similar non-Hispanic White cisgender beneficiaries. Overall, 6.3% of the excess peripheral vascular disease among Black TGD beneficiaries and 19.9% of the excess peripheral vascular disease among Asian and Pacific Islander TGD beneficiaries were associated with being at the intersection of gender, race, and ethnicity.

**Conclusions and Relevance:**

This cross-sectional study found that Asian and Pacific Islander, Black, and Hispanic TGD beneficiaries had a high prevalence of cardiovascular-related conditions and had an elevated prevalence of several conditions, attributable to the intersection of gender, race, and ethnicity. Medicare should use the tools at its disposal to support the health of TGD beneficiaries.

## Key Points

**Question:**

How does the prevalence of cardiovascular-related conditions differ among transgender and gender diverse (TGD) Medicare beneficiaries across racial and ethnic groups compared with cisgender beneficiaries?

**Findings:**

This repeated cross-sectional analysis that included 359 617 Medicare beneficiaries found that Asian and Pacific Islander and Black TGD beneficiaries had an excess prevalence of several cardiovascular-related conditions that were at the intersection of gender, race, and ethnicity.

**Meaning:**

TGD Asian and Pacific Islander, Black, and Hispanic beneficiaries experienced higher than expected rates of cardiovascular conditions based on rates of cardiovascular conditions among TGD White beneficiaries and cisgender beneficiaries of the same race.

## Teaser

This cross-sectional study compares the prevalence of cardiovascular-related conditions across racial and ethnic groups for transgender, gender-diverse, and cisgender people using quantitative intersectional methods.

## Introduction

Cardiovascular conditions are the leading cause of death in the US. In 2022, about 1 in 5 deaths in the US was attributable to cardiovascular diseases (CVDs).^1^ Transgender and gender diverse (TGD) people have higher rates of CVDs and their risk factors, including congestive heart failure, chronic kidney disease, hypertension, chronic obstructive pulmonary disease, and diabetes, than cisgender beneficiaries.^2,3,4^ These disparities derive from multiple sources including stigma, which induces stress and restricts access to the necessary resources needed to maintain one’s health.^4^ Stigma is the social process of labeling, stereotyping, and rejecting human differences as a form of social control.^5^ TGD-specific stigma can be structural (eg, laws that limit trans people’s access to public spaces), interpersonal (eg, employment discrimination), and individual (eg, health care avoidance due to anticipated discrimination).^6^

TGD people, who are multiply marginalized, are at even higher risk of adverse health outcomes. Indeed, an abundance of research highlights that Black and Latina TGD women are more likely to be living with HIV than White TGD women.^7,8^ These heightened disparities are at the intersection of TGD stigma and racism.^9,10^ A quantitative intersectional analysis found that between 50% and 85% of the excess risk of HIV in American Indian, Alaska Native, Black, Latinx, and Native Hawaiian and Pacific Islander transgender women was attributed to the intersection of gender, race, and ethnicity.^7^ Although CVDs are the leading cause of death, and substantial racial and ethnic disparities exist in the US,^11,12^ limited research has documented CVD differences across different racial and ethnic subgroups of TGD people or relative to their cisgender peers.

Intersectionality is a framework that investigates how interlocking systems of power impact the lived experiences of historically and socially marginalized groups.^13^ A key principle of intersectionality is that social categories like gender, race, and ethnicity are socially constructed, interconnected, and mutually influential.^14^ Over the past decade, methods have developed to integrate the core tenets of intersectionality into quantitative public health research.^15,16^ Despite these developments, to our knowledge, only one analysis^7^ has used quantitative intersectional methods to explore health disparities by gender modality^17^ (ie, the relationship between a person’s gender identity and gender assigned at birth such as transgender or cisgender), race, and ethnicity. Novel statistical methods and the availability of new algorithms to identify TGD people in claims data provide an opportunity to quantitatively explore the intersection of race and gender on CVD-related outcomes in non-Hispanic Asian and Pacific Islander, non-Hispanic Black, Hispanic, and non-Hispanic White TGD and cisgender people.

To address these research gaps, we used national Medicare data to compare the prevalence of CVDs and risk factors for CVD-related conditions across racial groups for TGD and cisgender people and employed quantitative intersectional methods to understand the complex effects of these intersecting identities on CVD-related conditions. We hypothesize that Asian and Pacific Islander, Black, and Hispanic TGD beneficiaries would have higher rates of CVD-related conditions than would be expected based on rates of conditions among White TGD beneficiaries and beneficiaries of the same race and ethnicity. Findings have implications for public health practice and the Medicare program.

## Methods

### Positionality

This team of lesbian, bisexual, and heterosexual researchers is composed of Black and White non-Hispanic people who identify as transgender men, cisgender women, and cisgender men. None of the authors identify as both transgender and Black. Our identities and perspectives informed the research questions that we asked, the analytical decisions we made, and our interpretation of our findings.

### Data Source

We used Medicare fee-for-service claims from 2011 to 2020 for this analysis. Our primary sources were the Medicare Master Beneficiary Summary File, 27 and 30 Chronic Condition Warehouse Chronic Conditions Segments, and Other Chronic or Potentially Disabling Conditions segments. We identified beneficiaries who became part of the TGD cohort using 100% files. Cisgender matches were identified from 20% of all fee-for-service Medicare beneficiaries. The Institutional Review Board of Brown University determined the study was exempt and waived patient consent. This study follows the Strengthening the Reporting of Observational Studies in Epidemiology (STROBE) reporting guidelines.

### Cohort

We identified likely TGD beneficiaries using an established algorithm^18^ that leverages diagnosis, prescription drug, and procedure codes from inpatient, outpatient, carrier (claims submitted by health care professionals), and Part D prescription claims. We then further classified beneficiaries into transmasculine and nonbinary and transfeminine and nonbinary groups. This algorithm has been found to have high sensitivity and specificity compared with self-reported gender.^19^ We considered beneficiaries who were not classified as TGD as cisgender. We treated gender modality (ie, TGD and cisgender) as time invariant because the effects of TGD stigma are unlikely to begin to affect beneficiaries when they are first categorized as TGD by the algorithm. The race and ethnicity variable in our analyses was classified using the Research Triangle International race code, which has 4 levels: non-Hispanic Asian and Pacific Islander, non-Hispanic Black, non-Hispanic White (henceforth Asian and Pacific Islander, Black, and White), and Hispanic. We excluded beneficiaries who were American Indian and Alaska Native or part of the other race category from our analyses due to limited sample sizes among the TGD cohort.

Beneficiaries must have had at least one inpatient, carrier, or outpatient claim between 2011 and 2020 to be included in this analysis. We excluded person-years in which beneficiaries were younger than 18 years old, had been Medicare beneficiaries for less than 2 years, lived outside of the 50 US states or Washington, DC, or had Medicare Advantage for the majority of the year.

### Outcomes

The primary outcomes in this analysis were CVD or risk factors defined as diabetes, hypertension, peripheral vascular disease, chronic obstructive pulmonary disease, and congestive heart failure. We defined each condition as a binary variable for which a beneficiary was considered having a condition if they met claims criteria set by the Chronic Conditions Warehouse.^20^

### Covariates

We determined beneficiary age, original basis of eligibility, years of Medicare enrollment, months of dual eligibility with Medicaid, months of Medicare Advantage coverage, and zip code from the Master Beneficiary Summary File. We categorized beneficiaries as dual eligible for Medicare and Medicaid if they were eligible for 6 or more months in a year. To account for variations in service intensity across the US, we assigned each beneficiary to a health services area using their zip code. All covariates were considered time varying except for beneficiaries’ original basis of eligibility.

### Statistical Analysis

We formatted the data as a repeated cross-sectional dataset on the person-year level. To account for observable differences between the TGD and cisgender cohorts, we estimated propensity scores based on age (continuous), years enrolled in Medicare (continuous), original basis of eligibility, race, ethnicity, and in the beneficiary’s final observable year. We then matched each TGD beneficiary to their 10 nearest neighbors in the cisgender cohort based on the propensity scores. Cohorts were considered balanced on a characteristic if they had a standardized mean difference (SMD) of 0.2 or less, based on previous literature.^21^

We fit generalized estimating equations modeling the prevalence of each condition of interest, controlling for original eligibility, number of years enrolled in Medicare, and age. We used a logit link and an autoregressive correlation structure over multiple years and clustered standard errors on the beneficiary. We report marginal estimates so that the intersection of each gender modality, race, and ethnicity is comparable. Then, we calculated the attributable proportion for each condition of interest using marginal estimates. The attributable proportion is the proportion of the mean outcome in a doubly marginalized group explained by the intersectional difference.^15,22^ For instance, for Hispanic TGD beneficiaries, the attributable difference for chronic obstructive pulmonary disease is the difference between the adjusted prevalence of chronic obstructive pulmonary disease for Hispanic TGD beneficiaries, Hispanic cisgender beneficiaries, and White TGD beneficiaries added to the prevalence for White cisgender beneficiaries. The attributable proportion is the attributable difference over the prevalence of chronic obstructive pulmonary disease for Hispanic TGD beneficiaries. Results are reported as significant if there is no overlap in 95% CIs.

In sensitivity analyses, we stratified our cohort by their original basis of Medicare eligibility because beneficiaries may have very different prevalences of disease outcomes of interest.^3^ We did not include beneficiaries who qualified based on end-stage renal disease in sensitivity analyses due to sample size. Because propensity scores were calculated to match cisgender and TGD beneficiaries, we report on racial and ethnic differences within TGD populations based on results that hold over both strata of beneficiaries qualified based on age and disability. We also report estimates for each condition category based on gender categorization (cisgender women, cisgender men, transfeminine and nonbinary, transmasculine and nonbinary, and TGD unclassified). Datasets were created in SAS version 9.4 (SAS Institute Inc), and all remaining analyses were conducted in Stata version 17 (StataCorp). These data were analyzed from November 7, 2023, to October 31, 2024.

## Results

We classified 36 693 TGD beneficiaries and 10 817 446 cisgender beneficiaries who were Asian and Pacific Islander, Black, Hispanic, or White. There were key demographic differences between TGD and cisgender people in each racial and ethnic category (eTable 1 in Supplement 1). As such, we calculated propensity scores based on age, years enrolled in Medicare, original basis of eligibility, race and ethnicity, and health services area. The propensity scores had substantial overlap. After matching, the cohort included 36 004 TGD and 323 613 cisgender beneficiaries, which were balanced (SMD ≤0.2) in each race and ethnicity subgroup (Table 1).

**Table 1.** Sample Characteristics by Race and Ethnicity and Gender Modality After Matching^a^

	Asian and Pacific Islander, No. (%)^b^			Black , No. (%)^b^			Hispanic, No. (%)			White, No. (%)^b^		
	Cisgender (n = 5981)	TGD (n = 714)	SMD^c^	Cisgender (n = 40 781)	TGD (n = 4518)	SMD^c^	Cisgender(n = 22 417)	TGD (n = 2545)	SMD^c^	Cisgender (n = 254 434)	TGD (n = 28 227)	SMD^c^
Age, mean (SD), y	65.0 (17.8)	64.6 (19.1)	NA	55.0 (17.8)	54.1 (18.3)	NA	56.8 (18.6)	57.9 (18.9)	NA	63.9 (17.5)	62.9 (18.3)	NA
Age category, y												
18-29	245 (4.1)	31 (4.3)	−0.04	3625 (8.9)	399 (8.8)	0.04	1979 (8.8)	218 (8.6)	−0.07	10 328 (4.1)	1675 (5.9)	0.04
30-44	800 (13.4)	111 (15.6)		9110 (22.3)	1172 (25.9)		4604 (20.5)	481 (18.9)		32 332 (12.7)	3750 (13.3)	
45-64	1115 (18.6)	143 (20.0)		14 117 (34.6)	1439 (31.9)		6649 (29.7)	760 (29.9)		65 540 (25.8)	6722 (23.8)	
65-84	3156 (52.8)	322 (45.1)		12 030 (29.5)	1278 (28.3)		7753 (34.6)	878 (34.5)		11 497 (46.6)	13 233 (46.9)	
≥85	665 (11.1)	107 (15.0)		1899 (4.7)	230 (5.1)		1432 (6.4)	208 (8.2)		27 737 (10.9)	2847 (10.1)	
Time enrolled in Medicare, mean (SD), y	12.6 (8.6)	13.6 (8.6)	−0.10	13.0 (9.4)	13.9 (9.1)	0.02	13.9 (10.0)	13.7 (9.0)	−0.11	15.2 (10.0)	14.7 (9.3)	0.05
Dual Medicare and Medicaid status												
Nondual	2912 (48.7)	319 (44.7)	−0.04	18 208 (44.7)	1607 (35.6)	−0.20	9579 (42.8)	960 (37.7)	−0.10	177 080 (69.6)	17 254 (61.1)	−0.17
Partial dual	247 (4.1)	48 (6.7)		4785 (11.7)	572 (12.7)		2151 (9.6)	240 (9.4)		16 087 (6.3)	2368 (8.4)	
Full dual	2821 (47.2)	347 (48.6)		17 796 (43.6)	2339 (51.8)		10 679 (47.7)	1345 (52.9)		61 191 (24.1)	8601 (30.5)	
Original entitlement reason												
Age	3551 (59.4)	387 (54.2)	−0.08	9620 (23.6)	1037 (23.0)	−0.04	7174 (32.0)	815 (32.0)	0.01	121 011 (47.6)	13 069 (46.3)	
Disability	2257 (37.7)	299 (41.9)		29 855 (73.2)	3277 (72.5)		14 572 (65.0)	1656 (65.1)		131 536 (51.7)	14 950 (53.0)	
End stage renal disease	173 (2.9)	28 (4.0)		1306 (3.2)	204 (4.5)		671 (3.0)	74 (3.0)		1887 (0.7)	208 (0.8)	
CMS region^d^												
Boston, Massachusetts	191 (3.2)	28 (3.9)	0.07	1121 (2.8)	132 (2.9)	−0.01	1405 (6.3)	174 (6.8)	0.03	21 146 (8.3)	2432 (8.6)	−0.02
New York, New York	880 (14.7)	113 (15.8)		4383 (10.8)	529 (11.7)		3385 (15.1)	427 (16.8)		23 722 (9.3)	2577 (9.1)	
Philadelphia, Pennsylvania	418 (7.0)	48 (6.7)		6420 (15.7)	709 (15.7)		784 (3.5)	97 (3.8)		26 267 (10.3)	2844 (10.1)	
Atlanta, Georgia	320 (5.4)	44 (6.2)		10 031 (24.6)	1048 (23.2)		2731 (12.2)	289 (11.4)		44 667 (17.6)	4730 (16.8)	
Chicago, Illinois	464 (7.8)	56 (7.8)		7494 (18.4)	760 (16.8)		1504 (6.7)	178 (7.0)		46 516 (18.3)	5118 (18.1)	
Dallas, Texas	255 (4.3)	39 (5.5)		5006 (12.3)	548 (12.1)		4582 (20.4)	494 (19.4)		22 712 (8.9)	2444 (8.7)	
Kansas City, Missouri	97 (1.6)	14 (2.0)		1924 (2.9)	128 (2.8)		279 (1.2)	42 (1.7)		14 298 (5.6)	1595 (5.6)	
Denver, Colorado	95 (1.6)	11 (1.5)		272 (0.7)	35 (0.8)		638 (2.9)	73 (2.9)		10 241 (4.0)	1192 (4.2)	
San Francisco, California	2906 (48.6)	315 (44.1)		4273 (10.5)	551 (12.2)		6514 (29.1)	687 (27.0)		29 110 (11.4)	3397 (12.0)	
Seattle, Washington	355 (5.9)	46 (6.4)		606 (1.5)	78 (1.7)		595 (2.7)	84 (3.3)		2906 (6.2)	1924 (6.8)	

Abbreviation: NA, not applicable.

^a^ Medicare claims data from 2011 to 2020 were used. Propensity scores are based on age, race and ethnicity, original basis of eligibility, years enrolled in Medicare, and health services area. Propensity scores to match each transgender and gender diverse (TGD) beneficiary to 10 nearest neighbors in the cisgender cohort. The mean (SD) for age and years enrolled in Medicare were reported.

^b^ Asian and Pacific Islander, Black, and White groups are all non-Hispanic.

^c^ Standardized mean differences (SMDs) were calculated by dividing the difference between cisgender and TGD beneficiaries by the estimate for cisgender beneficiaries. SMDs were considered to have no meaningful difference if the value were 0.2 or less.

^d^ Region data are presented using Centers for Medicare & Medicaid Services (CMS) regions, which are labeled with the location of the CMS regional office. All other variables are reported using column percentages.

### Gender Modality Comparison Within Racial and Ethnic Groups

In the matched populations, the most common CVD-related conditions were hypertension and diabetes for each racial, ethnic, and gender modality subgroup. TGD beneficiaries had a significantly higher risk of peripheral vascular disease, congestive heart failure, and chronic obstructive pulmonary disease than did cisgender beneficiaries of the same race and ethnicity (Table 2, Figure). Black, Hispanic, and White TGD beneficiaries had a higher risk of hypertension than did cisgender beneficiaries of the same race and ethnicity. White TGD beneficiaries had a higher risk of diabetes (marginal estimate, 30.8%; 95% CI, 30.3%-31.3%) than White cisgender beneficiaries (marginal estimate, 28.1%; 95% CI, 27.9%-28.3%).

**Table 2.** Marginal Estimates of Cardiovascular-Related Conditions by Racial, Ethnic, and Gender Modality^a^

Condition	Prevalence (95% CI), %^b^							
	Asian and Pacific Islander^c^		Black^c^		Hispanic		White^c^	
	Cisgender	TGD	Cisgender	TGD	Cisgender	TGD	Cisgender	TGD
PVD	12.5 (11.7-13.1)	19.6 (17.6-21.6)	17.5 (17.2-17.8)	22.1 (21.3-22.9)	17.2 (16.8-17.6)	20.0 (19.0-21.1)	12.7 (12.6-12.8)	15.9 (15.6-16.2)
CHF	15.8 (15.1-16.6)	22.0 (19.9-24.1)	24.0 (23.7-24.2)	27.0 (26.1-27.8)	19.5 (19.0-19.9)	21.7 (20.1-22.8)	15.9 (15.8-16.0)	18.8 (18.5-19.1)
Diabetes	42.8 (41.7-43.9)	44.4 (41.4-47.5)	41.6 (41.2-42.0)	42.2 (40.9-43.4)	43.1 (42.5-43.6)	43.5 (42.0-45.1)	28.1 (27.9-28.3)	30.8 (30.3-31.3)
Hypertension	54.9 (54.0-55.9)	58.5 (55.8-61.1)	65.7 (65.3-66.1)	67.6 (66.5-68.7)	56.3 (55.8-56.8)	59.8 (58.4-61.2)	49.8 (49.6-49.9)	54.1 (53.6-54.5)
COPD	9.5 (8.8-10.2)	14.9 (13.0-16.8)	13.4 (13.1-13.7)	17.2 (16.5-18.0)	11.3 (11.0-11.7)	16.1 (15.1-17.1)	13.9 (13.8-14.0)	18.1 (17.8-18.4)

Abbreviations: CHF, congestive heart failure; COPD, chronic obstructive pulmonary disease; PVD, peripheral vascular disease; TGD, transgender and gender diverse.

^a^ Medicare claims data were from 2011 to 2020. Propensity scores were based on age, race and ethnicity, original basis of eligibility, years enrolled in Medicare, and health services area. Propensity scores were used to match each TGD beneficiary to 10 nearest neighbors in the cisgender cohort. Marginal effects were estimated using generalized estimating equations with a logit link, controlling for original eligibility, year enrolled, race and ethnicity, and age, clustering standard errors on the individual and using an autoregressive correlation structure.

^b^ Adjusted prevalences include 95% CIs.

^c^ Asian and Pacific Islander, Black, and White groups are all non-Hispanic. Estimates stratified by original basis of eligibility are presented in eTables 3 and 4 in Supplement 1.

**Figure.**
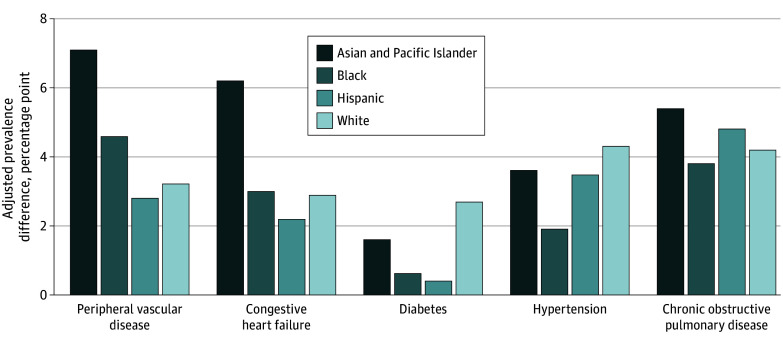
Differences in Adjusted Prevalences of Cardiovascular-Related Conditions Matched Between Cisgender and Transgender and Gender-Diverse (TGD) Medicare Beneficiaries by Race and Ethnicity After calculating the prevalences of cardiovascular-related conditions between cisgender and TGD Medicare beneficiaries adjusted for original eligibility, year enrolled, race and ethnicity, and age, we calculated the difference between TGD and cisgender adjusted prevalences for each race and ethnicity group.

Asian and Pacific Islander beneficiaries had the largest absolute differences between gender modalities of any racial and ethnic group. Asian and Pacific Islander TGD beneficiaries had a higher prevalence of peripheral vascular disease by 7.1 (95% CI, 5.0 to 9.2) percentage points, congestive heart failure by 6.2 (95% CI, 4.0 to 8.5) percentage points, chronic obstructive pulmonary disease by 5.4 percentage (95% CI, 4.4 to 6.4) points, hypertension by 3.6 percentage points (95% CI, 2.2 to 5.0), and diabetes by 1.6 percentage points (95% CI, −0.1 to 3.3) than Asian and Pacific Islander cisgender beneficiaries (Table 2).

Among the TGD subgroups, Asian and Pacific Islander transfeminine and nonbinary beneficiaries had a higher prevalence of chronic obstructive pulmonary disease (16.2%; 95% CI, 13.1%-19.3%; eTable 2 in Supplement 1) than cisgender men (11.9%; 95% CI, 10.9%-12.9%) or cisgender women (7.4%; 95% CI, 6.4%-8.4%). All Black TGD subgroups had a higher prevalence of peripheral vascular disease and chronic obstructive pulmonary disease than cisgender men and cisgender women. Black transmasculine and nonbinary beneficiaries had a higher prevalence of congestive heart failure than Black cisgender men, cisgender women, transfeminine, and nonbinary beneficiaries (28.9%; 95% CI, 27.4%-30.4%). Hispanic transfeminine and nonbinary and transmasculine and nonbinary beneficiaries had a higher prevalence of hypertension and chronic obstructive pulmonary disease than Hispanic cisgender women and cisgender men in the analysis. White transfeminine and nonbinary and transmasculine and nonbinary beneficiaries had a higher prevalence of all the outcomes of interest than White cisgender men and women. White transfeminine and nonbinary beneficiaries had a significantly higher rate of diabetes than any other gender category (32.5%; 95% CI, 31.8%-33.3%), and White transmasculine and nonbinary beneficiaries had a higher rate of chronic obstructive pulmonary disease than any other gender category (19.4%; 95% CI, 18.9%-20.9%).

### Comparison for All TGD Beneficiaries by Race and Ethnicity

Black TGD beneficiaries who qualified based on age had a higher prevalence of peripheral vascular disease than Asian and Pacific Islander as well as White TGD beneficiaries among beneficiaries who qualified based on age (eTable 3 in Supplement 1) and based on disability (eTable 4 in Supplement 1). Black TGD beneficiaries also had a higher prevalence of congestive heart failure than Asian and Pacific Islander in addition to White beneficiaries in both strata. Compared with White TGD beneficiaries, Black and Hispanic TGD beneficiaries had a higher prevalence of diabetes and hypertension. Among beneficiaries who qualified based on age, Asian and Pacific Islander TGD beneficiaries had a higher prevalence of diabetes (55.6%; 95% CI, 51.2%-59.9%) and hypertension (78.0%; 95% CI, 74.3%-81.6%) than White beneficiaries (32.6%; 95% CI, 31.8%-33.4% and 68.4%; 95% CI, 67.8%-69.0%, respectively; eTable 3 in Supplement 1).

### Joint Associations

Asian and Pacific Islander, Black, and Hispanic TGD beneficiaries had negative attributable proportions for diabetes (range, –2.5% to –5.3%) and hypertension (range, –1.2 to –3.6%; Table 3). For these conditions, there was a slightly lower risk than expected for Asian and Pacific Islander, Black, and Hispanic TGD beneficiaries compared with Asian and Pacific Islander, Black, and Hispanic cisgender, White cisgender, and White TGD beneficiaries. Black TGD beneficiaries had 74% higher prevalence of peripheral vascular disease, 76% higher prevalence of congestive heart failure, and 50% higher prevalence of diabetes than similar non-Hispanic White cisgender beneficiaries. We estimate that 6.3% of the excess peripheral vascular disease among Black TGD beneficiaries and 19.9% of the excess peripheral vascular disease among Asian and Pacific Islander TGD beneficiaries was at the intersection of gender, race, and ethnicity. Similarly, we estimated that 15.0% of the excess congestive heart failure among Asian and Pacific Islander TGD beneficiaries was attributable to the intersection of gender, race, and ethnicity. Among TGD Asian and Pacific Islander beneficiaries, 8.1% of the excess prevalence of chronic obstructive pulmonary disease is at the intersection of being Asian and Pacific Islander and being TGD.

**Table 3.** Attributable Proportion and Absolute Differences Between Racial and Ethnic and Gender Modality Subgroups^a^

Condition	Percentage point^b^			**Attributable proportion (Asian Pacific Islander TGD), %**
**Asian and Pacific Islander** ^c^	**Asian and Pacific Islander cisgender, White cisgender difference**	**Asian and Pacific Islander TGD, White TGD difference**	**Asian and Pacific Islander TGD, White cisgender difference**
PVD	−0.2	3.7	6.9	19.9
CHF	−0.1	3.2	6.1	15.0
Diabetes	14.7	13.6	16.3	−2.5
Hypertension	5.1	4.4	8.7	−1.2
COPD	−4.4	−3.2	1.0	8.1
**Black** ^c^	**Black cisgender, White cisgender difference**	**Black TGD, White TGD difference**	**Black TGD, White cisgender difference**	**Attributable proportion (Black TGD), %**
PVD	4.8	6.2	9.4	6.3
CHF	8.1	8.2	11.1	0.4
Diabetes	13.5	11.4	14.1	−5.0
Hypertension	15.9	13.5	17.8	−3.6
COPD	−0.5	−0.9	3.3	−2.3
**Hispanic** ^c^	**Hispanic cisgender, White cisgender difference**	**Hispanic TGD, White TGD difference**	**Hispanic TGD, White cisgender difference**	**Attributable proportion (Hispanic TGD), %**
PVD	4.5	4.1	7.3	−2.0
CHF	3.6	2.9	6.8	−3.2
Diabetes	15.0	12.7	15.4	−5.3
Hypertension	6.5	5.7	10.0	−1.3
COPD	−2.6	−2.0	2.2	3.7

Abbreviations: CHF, congestive heart failure; COPD, chronic obstructive pulmonary disease; PVD, peripheral vascular disease; TGD, transgender and gender diverse.

^a^ Medicare claims data were from 2011 to 2020. Propensity scores were based on age, race and ethnicity, original basis of eligibility, years enrolled in Medicare, and health services area. Propensity scores were used to match each transgender and gender diverse (TGD) beneficiary to 10 nearest neighbors in the cisgender cohort. We estimated marginal effects using generalized estimating equations with a logit link, controlling for original eligibility, year enrolled, race and ethnicity, and age, clustering standard errors on the individual and using an autoregressive correlation structure.

^b^ Asian and Pacific Islander, Black, and White groups are all non-Hispanic.

^c^ For Asian and Pacific Islander, Black, and Hispanic groups, (1) the absolute difference in marginal estimates between cisgender beneficiaries and White cisgender beneficiaries, (2) the absolute difference in marginal estimates between TGD beneficiaries and White TGD beneficiaries, (3) the absolute difference in marginal estimates between TGD beneficiaries and White cisgender beneficiaries, and (4) the attributable proportion for the TGD group. Measures 1 through 3 are reported as percentage point differences.

## Discussion

This is the first study, to our knowledge, to compare the prevalence of CVD-related conditions by race, ethnicity, and gender modality. We found that TGD beneficiaries had a high prevalence of every condition compared with cisgender beneficiaries of the same race and ethnicity. Asian and Pacific Islander as well as Black TGD beneficiaries had an elevated prevalence of several conditions, attributable to the intersection of gender, race, and ethnicity. Findings extend prior survey-based research documenting elevated CVD-related health risks of people who hold multiple marginalized identities. For instance, Black and Hispanic sexual minority women have worse cardiovascular health than Black and Hispanic heterosexual women.^23^

Our findings support a growing body of research^2,3,24^ that demonstrates that TGD people face substantial health disparities—not only in HIV and mental health but also in a number of previously understudied conditions, including hypertension, chronic kidney disease, chronic obstructive pulmonary disease, and heart failure. This study adds to this work by illustrating that there are also major racial disparities in these conditions within TGD populations. Structural racism and cissexism chronically trigger stress responses among TGD racial and ethnic minority groups and this may lead to early health deterioration in the form of weathering.^25^ The weathering was initially introduced to explain Black-White health disparities in the US^25^ and has since been extended to disparities based on gender modality.^26,27^ For instance, TGD people have a higher allostatic load than cisgender people, which is correlated with higher perceived stress, anxiety, depression, and lower quality of life.^28^

We also found signals of the excess prevalence of CVD-related conditions among Asian and Pacific Islander and Black TGD beneficiaries. This research adds to the mounting literature that describes poor health care access and poor health outcomes for TGD racial and ethnic minority groups, who experience multiple barriers to receiving high-quality health care. For instance, they are more likely to report anti-TGD discrimination in health care settings than White, non-Hispanic TGD people.^29^ Furthermore, TGD racial and ethnic minority groups are often unable to find a health care provider who meets their needs and preferences related to both gender identity and race and ethnicity,^30,31^ and they experience intersectional, structural stigma, which contributes to CVD risk. In a large survey, more than a third of Black, Hispanic, and Asian and Pacific Islander TGD respondents were living in poverty, which was higher than the poverty rate for overall TGD respondents (29%).^32^ This rate was nearly 3 times higher than the poverty rate in the US population overall (12%).^33,34^ Poverty contributes to adverse CVD outcomes including through high stress, environmental cardiovascular risk factors such as air and noise pollution, lack of access to healthful foods, and lack of access to parks and safe spaces to exercise.^35^

### Future Directions

To our knowledge, this is the first time quantitative intersectional methods have been used to explore gender modality and race and ethnicity using claims data. Algorithms that identify TGD people in claims data have expanded possibilities for TGD research in the US because claims data offer large sample sizes compared with other data sources. This research highlights new avenues of TGD research across disease states and types of health services. The TGD population in the US is heterogeneous, and intersectional research with TGD populations is critical for identifying and addressing inequities, particularly for TGD people living at the intersection of multiple systems of marginalization. Furthermore, future research using qualitative and mixed methods is needed to explore the risk and protective factors for TGD racial and ethnic minority groups that shape the patterns observed herein.

### Public Health Implications

We have documented substantial health disparities for TGD populations compared with cisgender populations and within TGD communities. The Medicare program may not be adequately equipped to tackle the challenges faced by TGD beneficiaries, particularly TGD beneficiaries in racial and ethnic minority groups. Medicare does not collect racial and ethnic^36^ or gender identity data^37^ from all beneficiaries, which creates substantial barriers to studying racial, gender modality, and intersectional disparities. Medicare should use the tools at its disposal to support the health of TGD beneficiaries, particularly TGD ethnic and minority groups. Increased monitoring stratified by gender modality, race, and ethnicity may be necessary within the Medicare Advantage program and value-based programs to ensure that TGD minority communities receive the attention they need because Black and Hispanic beneficiaries have often been overlooked.^38,39^ This research also has implications beyond the Medicare program, contributing to a growing literature that demonstrates that the health of TGD beneficiaries in racial and ethnic minority groups is affected by structural racism and cissexism. Further work should examine political and structural interventions to improve cardiovascular health among TGD racial and ethnic minority groups.

### Limitations

There are several limitations to this analysis. First, we do not know beneficiaries’ gender identities because Medicare does not collect self-reported gender identity. However, TGD identification and stratification algorithms have been found to have a high sensitivity and specificity compared with self-reported gender identity in the electronic health record, which is considered the criterion standard.^19^ Second, current Medicare race and ethnicity misclassifies and biases outcomes, particularly for American Indian, Alaska Native, Asian, Hispanic, Native Hawaiian, Pacific Islander, and multiracial beneficiaries. We used the Research Triangle International race code for this analysis, which is more complete than the Medicare Enrollment Database race code.^36^ However, the Research Triangle International race variable has major limitations, including combining Asian and Pacific Islander categories. Third, we use race, ethnicity, and gender modality as proxies for racism and cissexism because we do not directly measure these constructs. Fourth, this work only includes fee-for-service Medicare beneficiaries and is, therefore, not generalizable to the full Medicare population or the US population. Future work should also include Medicare Advantage, given the large number of Asian, Black, Hispanic, and Pacific Islander beneficiaries in Medicare Advantage.^40^ Fifth, this descriptive analysis does not use causal inference methods. Finally, both the algorithm that identifies beneficiaries as TGD and the Chronic Conditions Warehouse disease definitions are utilization based. To limit potential confounding, we limited our analyses to beneficiaries who had utilization over the study period.

## Conclusions

This cross-sectional study found that Asian and Pacific Islander, Black, and Hispanic TGD beneficiaries had a high prevalence of CVD-related conditions. Asian and Pacific Islander, Black, and Hispanic TGD beneficiaries had an elevated prevalence of several conditions, attributable to the intersection of gender, race, and ethnicity. Medicare should use the tools at its disposal to support the health of TGD beneficiaries.

## Notes

Supplement 1. **eTable 1.** Sample Characteristics by Race and Ethnicity and Gender Modality Before Matching **eTable 2.** Marginal Estimates of Cardiovascular-Related Conditions by Race and Ethnicity and Gender Category **eTable 3.** Marginal Estimates of Cardiovascular-Related Conditions by Race and Ethnicity and Gender Modality Among Beneficiaries Originally Entitled by Age **eTable 4.** Marginal Estimates of Cardiovascular-Related Conditions by Race and Ethnicity and Gender Modality Among Beneficiaries Originally Entitled by Disability **eFigure 1.** Adjusted Prevalence of Peripheral Vascular Disease for Medicare Beneficiaries by Gender Modality and Race/Ethnicity, 2011-2020 **eFigure 2.** Adjusted Prevalence of Congestive Heart Failure for Medicare Beneficiaries by Gender modality and Race/Ethnicity, 2011-2020 **eFigure 3.** Adjusted Prevalence of Diabetes for Medicare Beneficiaries by Gender Modality and Race/Ethnicity, 2011-2020 **eFigure 4.** Adjusted Prevalence of Hypertension for Medicare Beneficiaries by Gender Modality and Race/Ethnicity, 2011-2020 **eFigure 5.** Adjusted Prevalence of Chronic Obstructive Pulmonary Disease for Medicare Beneficiaries by Gender Modality and Race/Ethnicity, 2011-2020

Supplement 2.
Data Sharing Statement
