# Supplementary material for: Cardiovascular Health at the Intersection of Race and Gender in Medicare Fee for Service
Source: JAMA Health Forum. 2025 Aug 22;6(8):e253014. doi: 10.1001/jamahealthforum.2025.3014 (PMC12374219; doi:10.1001/jamahealthforum.2025.3014)
Supplement: Supplement 1. — eTable 1. Sample Characteristics by Race and Ethnicity and Gender Modality Before Matching eTable 2. Marginal Estimates of Cardiovascular-Related Conditions by Race and Ethnicity and Gender Category eTable 3. Marginal Estimates of Cardiovascular-Related Conditions by Race and Ethnicity and Gender Modality Among Beneficiaries Originally Entitled by Age eTable 4. Marginal Estimates of Cardiovascular-Related Conditions by Race and Ethnicity and Gender Modality Among Beneficiaries Originally Entitled by Disability eFigure 1. Adjusted Prevalence of Peripheral Vascular Disease for Medicare Beneficiaries by Gender Modality and Race/Ethnicity, 2011-2020 eFigure 2. Adjusted Prevalence of Congestive Heart Failure for Medicare Beneficiaries by Gender modality and Race/Ethnicity, 2011-2020 eFigure 3. Adjusted Prevalence of Diabetes for Medicare Beneficiaries by Gender Modality and Race/Ethnicity, 2011-2020 eFigure 4. Adjusted Prevalence of Hypertension for Medicare Beneficiaries by Gender Modality and Race/Ethnicity, 2011-2020 eFigure 5. Adjusted Prevalence of Chronic Obstructive Pulmonary Disease for Medicare Beneficiaries by Gender Modality and Race/Ethnicity, 2011-2020 [file jamahealthforum-e253014-s001.pdf]

Supplemental Online Content

Babbs G, Offiaeli K, Hughto JMW, Hughes LD, Shireman TI, Meyers DJ.Cardiovascular health at the intersection of race and gender in Medicare fee for service. *JAMA Health Forum*. Published online August 22, 2025.
doi:10.1001/jamahealthforum.2025.3014

eTable 1. Sample Characteristics by Race and Ethnicity and Gender Modality Before Matching
eTable 2. Marginal Estimates of Cardiovascular-Related Conditions by Race and Ethnicity and Gender Category
eTable 3. Marginal Estimates of Cardiovascular-Related Conditions by Race and Ethnicity and Gender Modality Among Beneficiaries Originally Entitled by Age
eTable 4. Marginal Estimates of Cardiovascular-Related Conditions by Race and Ethnicity and Gender Modality Among Beneficiaries Originally Entitled by Disability
eFigure 1. Adjusted Prevalenceof Peripheral Vascular Disease for Medicare Beneficiaries by Gender Modality and Race/Ethnicity, 2011-2020
eFigure 2. Adjusted Prevalence of Congestive Heart Failure for Medicare Beneficiaries by Gender modality and Race/Ethnicity, 2011-2020
eFigure 3. Adjusted Prevalence of Diabetes for Medicare Beneficiaries by Gender Modlity and Race/Ethnicity, 2011-2020
eFigure 4. Adjusted Prevalence of Hypertension for Medicare Beneficiaries by Gender Modlity and Race/Ethnicity, 2011-2020
eFigure 5. Adjusted Prevalence of Chronic Obstructive Pulmonary Disease for Medicare Beneficiaries by Gender Modlity and Race/Ethnicity, 2011-2020

This supplemental material has been provided by the authors to give readers additional information about their work.

© 2025 Babbs G et al. *JAMA Health Forum*.

eTable 1. Sample Characteristics by Race and Ethnicity and Gender Modality Before Matching

	Cisgender	TGD	SMD	Cisgender	TGD	SMD	Cisgender	TGD	SMD	Cisgender	TGD	SMD
	N=310,208	N=725		N=1,131,635	N=4,582		N=730,707	N=2,551		N=8,644,896	N=28,835	
Age	72.9 (11.5)	63.6 (19.3)	0.58	66.9 (14.2)	53.1 (18.4)	0.84	68.6 (13.7)	56.8 (19.0)	0.71	72.9 (12.1)	62.0 (18.4)	0.70
Age Category			0.58			0.81			0.69			0.68
18-29	0.5%	5.8%		1.6%	11.4%		1.4%	9.8%		0.5%	6.7%	
30-44	2.0%	15.4%		6.0%	25.2%		4.9%	19.5%		2.2%	13.3%	
45-64	13.0%	21.2%		29.3%	32.4%		24.1%	31.1%		14.8%	25.6%	
65-84	68.6%	42.9%		53.1%	26.5%		58.4%	32.1%		65.0%	44.9%	
85+	15.8%	14.6%		10.0%	4.5%		11.3%	7.4%		17.6%	9.5%	
Years Enrolled in Medicare	10.7 (8.3)	11.9 (8.7)	-0.14	11.8 (9.5)	12.1 (8.9)	-0.04	10.8 (8.9)	12.1 (9.1)	-0.14	13.1 (9.4)	12.9 (9.4)	0.02
Original Basis of Eligibility			-0.70			-0.63			-0.65			-0.68
Age	85.9%	53.9%		54.9%	23.2%		65.7%	32.2%		78.5%	46.5%	
Disability	12.7%	42.2%		42.0%	72.4%		31.9%	65.0%		21.0%	52.7%	
End Stage Renal Disease	1.4%	3.9%		3.1%	4.4%		2.4%	2.9%		0.5%	0.7%	
Dual Eligibility			-0.24			-0.56			-0.40			-0.57
Non-Dual	58.5%	45.2%		64.2%	37.0%		57.2%	37.8%		86.3%	62.0%	
Partial Dual	3.2%	6.3%		9.0%	12.7%		7.8%	9.1%		3.4%	8.2%	
Full Dual	38.2%	48.3%		26.7%	50.3%		34.9%	53.0%		10.2%	29.8%	
Missing	0.1%	0.1%		0.1%	0.0%		0.1%	0.1%		0.1%	0.1%	
CMS Region			0.008			-0.13			0.08			-0.05
Region 1 - Boston	3.2%	4.0%		2.1%	2.9%		4.1%	7.1%		6.0%	8.6%	
Region 2 - New	13.1%	16.0%		9.6%	11.7%		11.8%	15.5%		8.1%	9.1%	

© 2025 Babbs G et al. *JAMA Health Forum.*

York												
Region 3 - Philadelphia	8.0%	6.9%		15.6%	15.6%		3.8%	4.0%		10.8%	10.1%	
Region 4 - Atlanta	7.5%	5.9%		31.7%	23.1%		13.7%	11.5%		21.0%	16.6%	
Region 5 - Chicago	8.8%	8.1%		16.8%	17.0%		7.4%	7.0%		19.3%	18.1%	
Region 6 - Dallas	7.2%	4.8%		13.9%	12.1%		24.4%	19.5%		10.9%	8.6%	
Region 7 - Kansas City	1.4%	1.9%		2.7%	2.8%		1.4%	1.6%		6.1%	5.5%	
Region 8 - Denver	1.4%	1.7%		0.5%	0.8%		2.7%	2.8%		3.8%	4.2%	
Region 9 - San Francisco	44.5%	44.3%		6.2%	12.2%		28.5%	27.4%		9.5%	12.3%	
Region 10 - Seattle	4.9%	6.3%		0.8%	1.7%		2.2%	3.5%		4.5%	6.9%	

Caption: Our study used Medicare claims data from 2011-2020. We reported mean and standard deviation (SD) for age and years enrolled in Medicare. Region data is presented using Centers for Medicare and Medicaid (CMS) regions. All other variables are reported using column percentages. We calculated standardized mean differences (SMD) by dividing the difference between cisgender and TGD beneficiaries by the estimate for cisgender beneficiaries. SMD were considered to have no meaningful difference if the value were 0.2 or less. Asian and Pacific Islander, Black, and White groups are all non-Hispanic.

© 2025 Babbs G et al. *JAMA Health Forum.*

eTable 2. Marginal Estimates of Cardiovascular-Related Conditions by Race and Ethnicity and Gender Category

Prevalence, % (95% CI)	Asian and Pacific Islander				
	Cisgender Men	Cisgender Women	TFN	TMN	TGD Unclassified
Peripheral Vascular Disease	13.5% (12.5-14.6)	11.4% (10.5-12.4)	19.7% (16.6-22.9)	21.0% (17.4-24.7)	18.1% (14.5-21.6)
Congestive Heart Failure	18.1% (16.9-19.2)	13.8% (12.8-14.9)	21.0% (17.7-24.4)	23.3% (19.4-27.2)	21.9% (18.1-25.7)
Diabetes	45.2% (43.6-46.8)	40.6% (39.1-42.1)	46.5% (41.7-51.2)	45.9% (40.5-51.3)	40.5% (35.0-46.1)
Hypertension	56.4% (55.0-57.8)	53.6% (55.5-64.1)	59.8% (55.5-64.1)	60.3% (55.5-65.2)	55.3% (50.6-60.0)
Chronic Obstructive Pulmonary Disease	11.9% (10.9-12.9)	7.4% (6.4-8.4)	16.2% (13.1-19.3)	16.3% (12.8-19.9)	12.0% (8.6-15.4)
	Black				
	Cisgender Men	Cisgender Women	TFN	TMN	TGD Unclassified
Peripheral Vascular Disease	17.9% (17.5-18.3)	17.2% (16.8-17.6)	21.5% (20.3-22.7)	22.6% (21.2-24.0)	22.6% (21.0-24.1)
Congestive Heart Failure	24.0% (23.6-24.4)	24.0% (23.6-24.4)	25.3% (24.0-26.6)	28.9% (27.4-30.4)	27.5% (25.9-29.2)
Diabetes	38.8% (38.1-39.4)	44.3% (43.7-44.9)	42.0% (40.0-43.9)	44.4% (42.1-46.6)	40.2% (37.7-42.6)
Hypertension	62.4% (61.8-62.9)	68.8% (68.3-69.3)	67.7% (66.0-69.4)	70.3% (68.3-72.2)	64.5% (62.4-66.5)
Chronic Obstructive Pulmonary Disease	13.5% (13.1-13.9)	13.3% (12.9-13.7)	17.4% (16.2-18.6)	18.3% (17.0-19.7)	15.8% (14.4-17.3)
	Hispanic				
	Cisgender Men	Cisgender Women	TFN	TMN	TGD Unclassified
Peripheral Vascular Disease	17.9% (17.4-18.5)	16.5% (16.0-17.1)	20.0% (18.4-21.5)	19.5% (17.6-21.4)	20.9% (18.8-23.1)
Congestive Heart Failure	20.3% (19.8-20.9)	18.6% (18.0-19.2)	20.4% (18.7-22.1)	21.6% (19.6-23.6)	24.5% (22.2-26.8)
Diabetes	43.9% (43.1-44.7)	42.2% (41.4-43.0)	44.3% (41.9-46.6)	43.4% (40.7-46.2)	42.7% (39.5-45.9)
Hypertension	56.5% (55.8-57.2)	56.1% (55.4-56.8)	59.9% (57.7-62.0)	60.4% (57.9-62.9)	59.0% (56.1-61.8)
Chronic Obstructive Pulmonary Disease	11.5% (11.0-12.0)	11.2% (10.7-11.7)	15.8% (14.3-17.4)	17.2% (15.4-19.0)	15.2% (13.2-17.3)
	White				
	Cisgender Men	Cisgender Women	TFN	TMN	TGD Unclassified

© 2025 Babbs G et al. *JAMA Health Forum.*

Peripheral Vascular Disease	13.7% (13.6-13.9)	11.7% (11.6-11.9)	15.9% (15.5-16.4)	15.7% (15.2-16.3)	16.2% (15.6-16.8)
Congestive Heart Failure	17.3% (17.1-17.5)	14.6% (14.5-14.8)	18.0% (17.5-18.5)	18.9% (18.3-19.5)	20.1% (19.4-20.8)
Diabetes	29.9% (29.7-30.2)	26.3% (26.1-26.5)	32.5% (31.8-33.3)	30.8% (30.0-31.7)	27.8% (26.8-28.8)
Hypertension	50.8% (50.6-51.0)	48.8% (48.6-49.0)	55.6% (55.0-56.3)	55.9% (55.1-56.6)	49.3% (48.5-50.2)
Chronic Obstructive Pulmonary Disease	14.0% (13.9-14.2)	13.8% (13.6-13.9)	17.7% (17.2-18.1)	19.4% (18.9-20.9)	17.2% (16.6-17.8)

Caption: Our study used Medicare claims data from 2011-2020. We estimated propensity scores based on age, race and ethnicity, original basis of eligibility, years enrolled in Medicare, and health services area. We used propensity scores to match each transgender and gender-diverse (TGD) beneficiary to 10 nearest neighbors in the cisgender cohort. We estimated marginal effects for 5 gender categories— cisgender men, cisgender women, transfeminine and non-binary (TFN), transmasculine and non-binary (TMN), and TGD unclassified— using generalized estimating equations with a logit link, controlling for original eligibility, year enrolled, race and ethnicity, and age, clustering standard errors on the individual and using an autoregressive correlation structure. Adjusted prevalences include 95% confidence intervals (CI). Asian and Pacific Islander, Black, and White groups are all non-Hispanic.

© 2025 Babbs G et al. *JAMA Health Forum.*

eTable 3. Marginal Estimates of Cardiovascular-Related Conditions by Race and Ethnicity and Gender Modality Among Beneficiaries Originally Entitled by Age

Prevalence, % (95% CI)	Asian and Pacific Islander		Black		Hispanic		White	
Condition	Cisgender	TGD	Cisgender	TGD	Cisgender	TGD	Cisgender	TGD
Peripheral Vascular Disease	17.3% (16.3-18.4)	28.5% (25.5-31.4)	24.3% (23.7-24.9)	38.0% (36.2-39.8)	24.2% (23.5-25.0)	30.3% (28.3-32.4)	16.4% (16.2-16.5)	22.3% (21.8-22.8)
Congestive Heart Failure	19.9% (18.8-21.0)	29.1% (26.0-32.2)	29.1% (28.4-29.7)	38.1% (36.2-40.0)	25.3% (24.5-26.1)	31.4% (29.2-33.5)	20.0% (19.8-20.2)	25.6% (25.1-26.1)
Diabetes	49.2% (47.7-50.7)	55.6% (51.2-59.9)	50.1% (49.2-51.0)	56.3% (53.6-59.0)	50.4% (49.3-51.4)	52.1% (49.2-55.0)	29.2% (28.9-29.4)	32.6% (31.8-33.4)
Hypertension	70.6% (69.4-71.9)	78.0% (74.3-81.6)	79.7% (78.9-80.4)	87.9% (85.6-90.1)	70.3% (69.5-71.2)	77.0% (74.5-79.5)	62.8% (62.6-63.0)	68.4% (67.8-69.0)
Chronic Obstructive Pulmonary Disease	10.7% (9.8-11.7)	19.1% (16.4-21.9)	15.0% (14.5-15.6)	22.9% (21.2-24.5)	14.1% (13.4-14.7)	21.7% (19.9-23.6)	13.7% (13.6-13.9)	20.0% (19.6-20.5)

Caption: Our study used Medicare claims data from 2011-2020. We estimated propensity scores based on age, race and ethnicity, original basis of eligibility, years enrolled in Medicare, and health services area. We used propensity scores to match each transgender and gender diverse (TGD) beneficiary to 10 nearest neighbors in the cisgender cohort. We estimated marginal effects using generalized estimating equations with a logit link, controlling for original eligibility, year enrolled, race and ethnicity, and age, clustering standard errors on the individual and using an autoregressive correlation structure. Here, we only present results for beneficiaries who originally qualify based on age (65+). Adjusted prevalences include 95% confidence intervals (CI). Asian and Pacific Islander, Black, and White groups are all non-Hispanic.

© 2025 Babbs G et al. *JAMA Health Forum.*

eTable 4. Marginal Estimates of Cardiovascular-Related Conditions by Race and Ethnicity and Gender Modality Among Beneficiaries Originally Entitled by Disability

Prevalence, % (95% CI)	Asian and Pacific Islander		Black		Hispanic		White	
Condition	Cisgender	TGD	Cisgender	TGD	Cisgender	TGD	Cisgender	TGD
Peripheral Vascular Disease	7.8% (6.8-8.8)	9.2% (6.4-11.9)	12.9% (12.6-13.2)	14.4% (13.6-15.2)	12.0% (11.6-12.4)	13.0% (11.9-14.2)	9.9% (9.7-10.0)	10.8% (10.4-11.1)
Congestive Heart Failure	12.4% (11.3-13.6)	13.8% (10.8-16.8)	19.4% (19.1-19.7)	19.8% (18.9-20.7)	14.5% (14.1-15.0)	14.7% (13.4-16.0)	12.5% (12.4-12.7)	13.1% (12.7-13.5)
Diabetes	34.8% (33.1-36.5)	31.8% (27.5-36.2)	37.6% (37.1-38.1)	36.8% (35.4-38.2)	38.5% (37.8-39.2)	38.3% (36.5-40.2)	26.7% (26.5-26.9)	28.7% (28.0-29.3)
Hypertension	40.3% (38.8-41.9)	39.9% (35.9-43.9)	54.5% (54.0-54.9)	54.7% (53.4-55.9)	44.6% (44.0-45.2)	46.7% (44.9-48.4)	38.8% (38.6-39.0)	42.0% (41.4-42.5)
Chronic Obstructive Pulmonary Disease	8.3% (7.2-9.4)	10.4% (7.5-13.3)	12.7% (12.4-13.1)	15.2% (14.3-16.1)	10.0% (9.6-10.5)	13.6% (12.4-14.8)	14.1% (14.0-14.3)	16.5% (16.0-16.9)

Caption: Our study used Medicare claims data from 2011-2020. We estimated propensity scores based on age, race and ethnicity, original basis of eligibility, years enrolled in Medicare, and health services area. We used propensity scores to match each transgender and gender-diverse (TGD) beneficiary to 10 nearest neighbors in the cisgender cohort. We estimated marginal effects using generalized estimating equations with a logit link, controlling for original eligibility, year enrolled, race and ethnicity, and age, clustering standard errors on the individual and using an autoregressive correlation structure. Here, we only present results for beneficiaries who originally qualify based on disability. Adjusted prevalences include 95% confidence intervals (CI). Asian and Pacific Islander, Black, and White groups are all non-Hispanic.

© 2025 Babbs G et al. *JAMA Health Forum.*

Please change the figure titles to eFigure

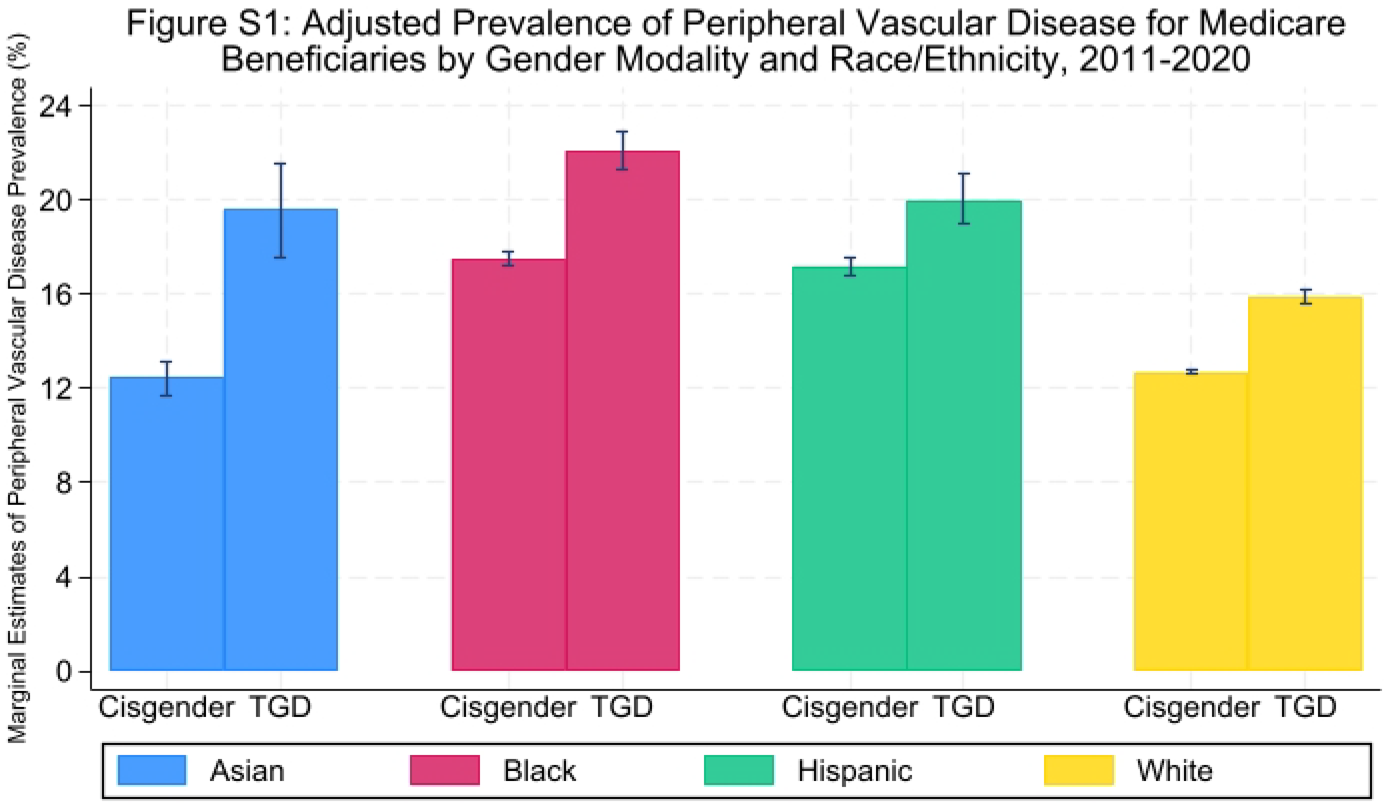

© 2025 Babbs G et al. *JAMA Health Forum*.

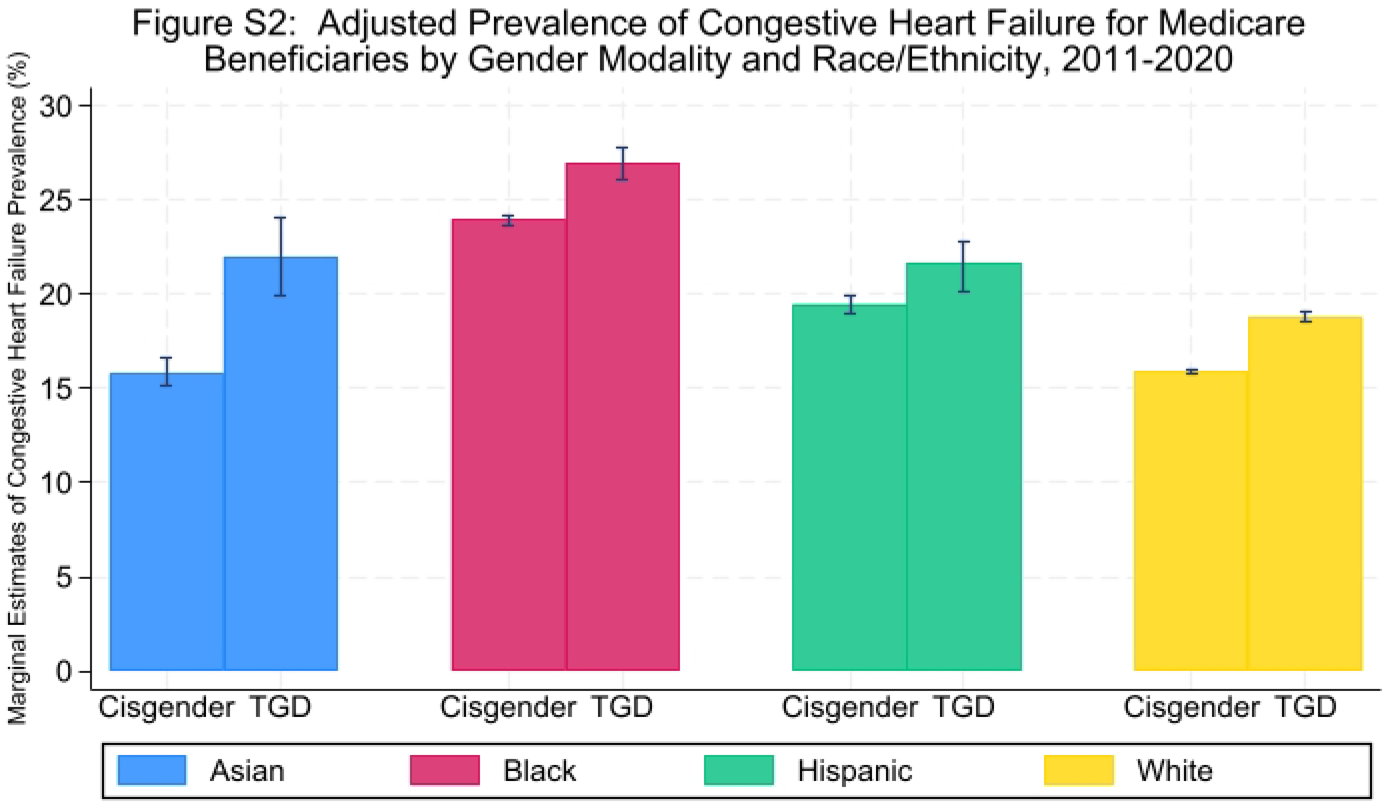

© 2025 Babbs G et al. *JAMA Health Forum*.

Figure S3: Adjusted Prevalence of Diabetes for Medicare Beneficiaries by Gender Modality and Race/Ethnicity, 2011-2020

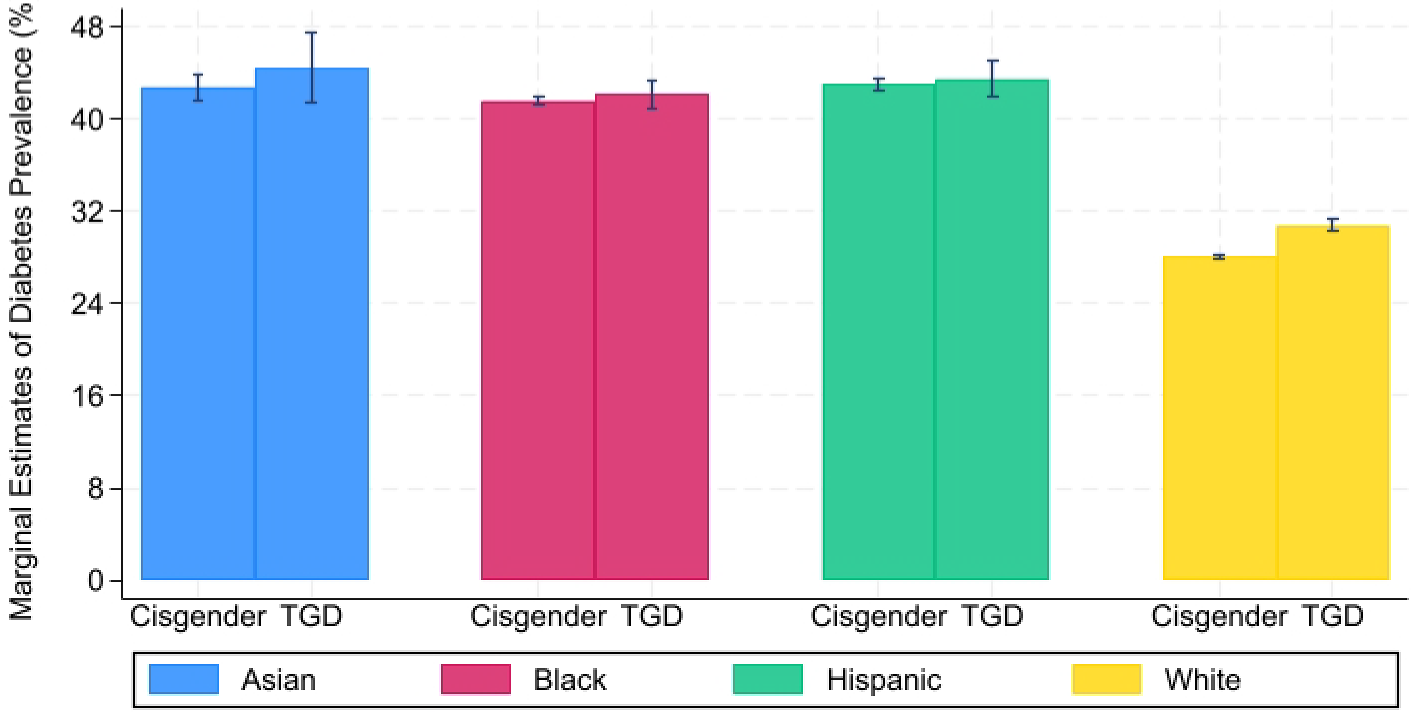

© 2025 Babbs G et al. *JAMA Health Forum*.

Figure S4: Adjusted Prevalence of Hypertension for Medicare Beneficiaries by Gender Modality and Race/Ethnicity, 2011-2020

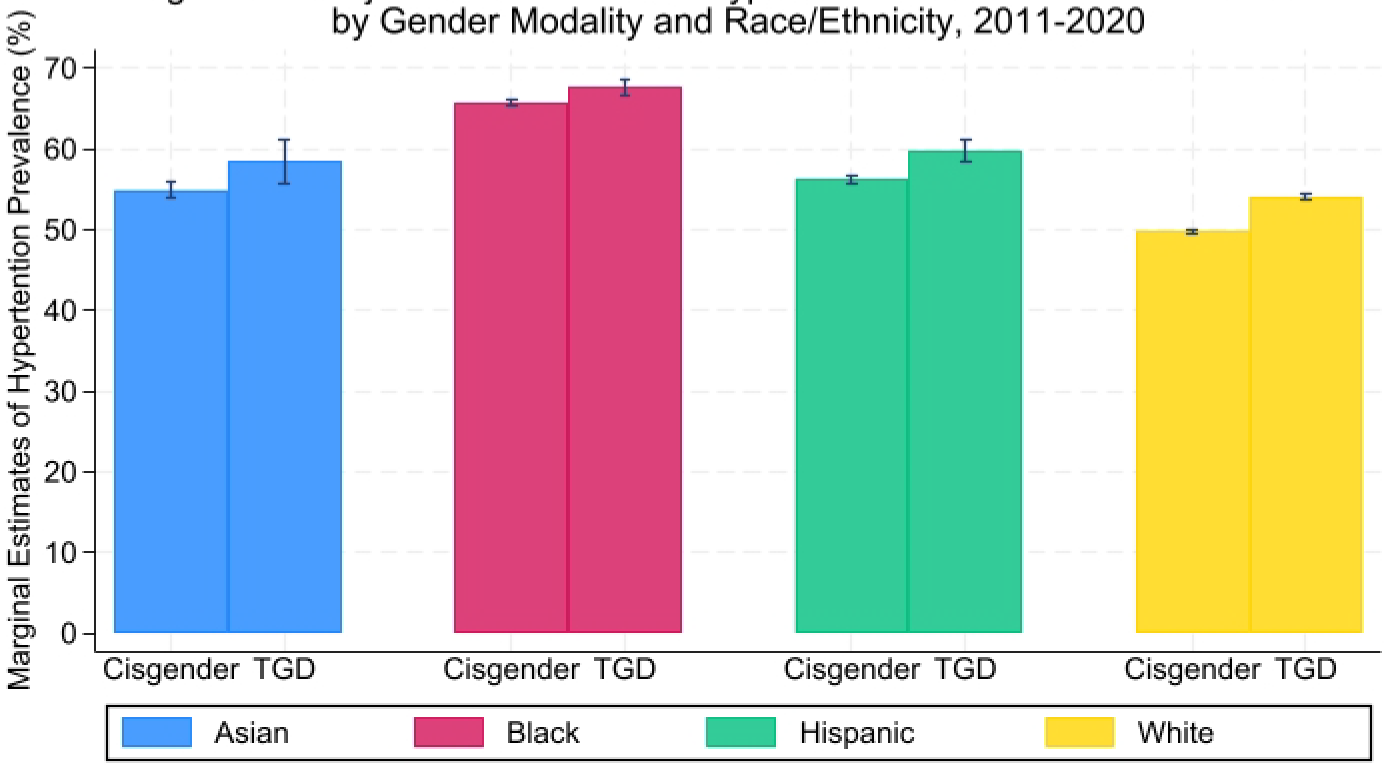

© 2025 Babbs G et al. *JAMA Health Forum*.

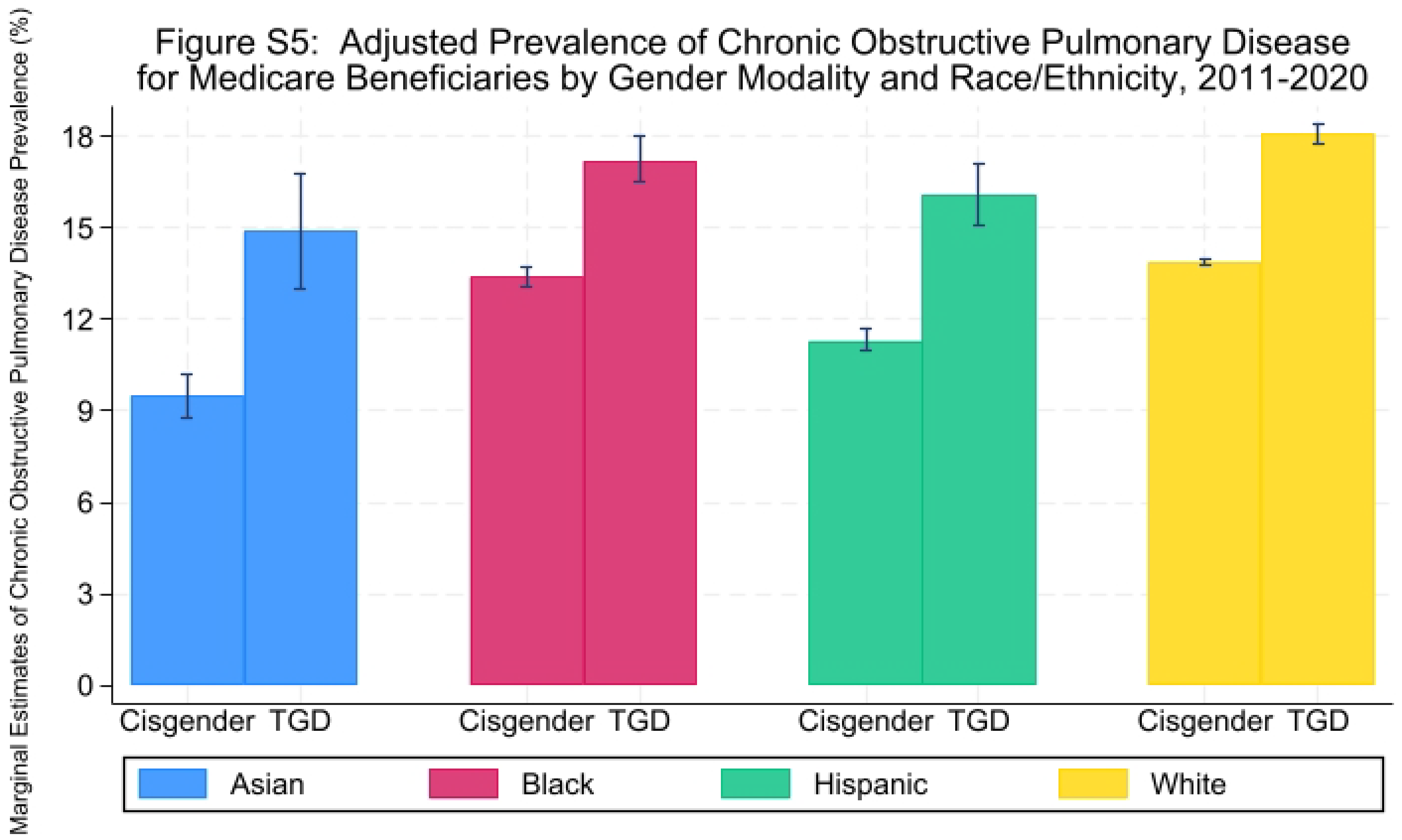

© 2025 Babbs G et al. *JAMA Health Forum*.
