# Supplementary material for: Cardiovascular Health at the Intersection of Race and Gender in Medicare Fee for Service
Source: JAMA Health Forum. 2025 Aug 22;6(8):e253014. doi: 10.1001/jamahealthforum.2025.3014 (PMC12374219; doi:10.1001/jamahealthforum.2025.3014)
Supplement: Supplement 2. — Data Sharing Statement [file jamahealthforum-e253014-s002.pdf]

Data Sharing Statement

Babbs. Cardiovascular Health at the Intersection of Race and Gender in Medicare Fee for Service. *JAMA Health Forum*. Published August 22, 2025.
doi:10.1001/jamahealthforum.2025.3014

Data
Data available: No

Additional Information
Explanation for why data not available: Data cannot be shared due to data sharing agreement. Data can be obtained through the Centers for Medicare and Medicaid Services.
